# Supplementary material for: Inactivation of NUPR1 promotes cell death by coupling ER-stress responses with necrosis
Source: Sci Rep. 2018 Nov 19;8:16999. doi: 10.1038/s41598-018-35020-3 (PMC6242935; doi:10.1038/s41598-018-35020-3)
Supplement: Supplementary file 1 — Supplementary data [file 41598_2018_35020_MOESM1_ESM.pdf]

Inactivation of NUPR1 promotes cell death by coupling ER-stress responses with necrosis

Patricia Santofimia-Castaño[1], Wenjun Lan[1], Jennifer Bintz[1], Odile Gayet[1], Alice Carrier[1], Gwen Lomberk[2], José Luis Neira[3], Antonio González[4], Raul Urrutia[2], Philippe Soubeyran[1], Juan Iovanna[1]

[1]Centre de Recherche en Cancérologie de Marseille (CRCM), INSERM U1068, CNRS UMR 7258, Aix-Marseille Université and Institut Paoli-Calmettes, Parc Scientifique et Technologique de Luminy, Marseille, France; [2]Division of Research, Department of Surgery and the Genomic Sciences and Precision Medicine Center, Medical College of Wisconsin, Milwaukee, USA ; [3]Instituto de Biología Molecular y Celular, Universidad Miguel Hernández, Edificio Torregaitán, Avda. del Ferrocarril s/n, 03202 Elche, Alicante, Spain; [4]Cell Physiology Research Group, Department of Physiology, University of Extremadura, Caceres, Spain.

Gene name	MiaPaCa2 cells siCtrl gene expression	MiaPaCa2 cells siNUPR1 gene expression
AMFR	61,49	26,99
AGR2	18,99	7,63
ASF1B	44,87	13,64
ATF4	75,49	18,29
ATF6	448,73	329,27
BCL2	253,12	8,66
CALM1	66,38	27,45
CALR	908,29	445,96
CANX	444,36	46,99
CAPS2	140,72	92,51
CCT6A	4131,92	1048,25
CCT6B	241,67	83,93
COPA	1760,16	1381,22
DDIT3	98,00	83,00
DNAJA1	15,04	5,10
DNAJA2	1726,94	854,32
DNAJA3	419,47	176,89
DNAJA4	56,87	8,66
DNAJB1	117,76	21,72
DNAJB11	2211,27	769,29
DNAJB14	260,37	129,23
DNAJB6	395,33	176,33
DNAJC10	319,00	178,00
DNAJC18	13,21	6,42
DNAJC19	662,03	509,57
DNAJC3	145,00	132,00
DNAJC6	633,32	54,62
DNAJC8	106,99	37,62
EDEM1	406,49	110,74
EDEM3	320,19	256,49
EIF1	1477,15	731,99
EIF1AX	202,56	88,85
EIF2A	52,67	22,53
EIF2AK2	344,52	123,95
EIF2AK3	120,00	97,00
EIF2B2	14,19	6,94
EIF2B4	386,37	186,87
EIF3A	31,87	12,62
EIF3D	817,16	319,18
EIF3E	246,46	93,16
EIF3H	182,84	50,99
EIF3J	979,17	485,99
EIF3K	183,10	88,74
EIF3M	257,77	115,81
ERN1	390,60	377,32
ERN2	413,76	319,32
ERO1A	470,22	352,10
ERO1B	396,96	144,13
FKBP1B	54,53	12,79
FKBP5	860,12	542,76
FKBP7	484,49	292,37
GRPEL2	33,48	11,34
HERPUD1	653,00	589,00
HERPUD2	13302,96	5390,39
HSP90AA1	154,39	52,99
HSP90AB1	7359,54	2546,68

HSP90B1	2234,00	1753,00
HSP90B3P	9,14	4,31
HSPA14	500,62	203,37
HSPA1A/HSPA1B	1351,64	445,24
HSPA4	3823,82	1267,70
HSPA4L	1645,63	757,66
HSPA5	836,86	188,99
HSPA8	3767,52	1766,77
HSPB2	1172,44	573,58
HSPB7	82,85	22,70
HSPB8	784,99	35,24
HSPBAP1	605,12	142,49
HSPH1	256,67	133,62
LMAN1	1074	844
MAN1A2	246,91	178,21
MAN2A1	269,93	236,98
MAN2B2	124,19	97,89
MANBA	84,44	70,59
NFE2L2	281,21	134,60
PDIA3	125,77	53,38
PPIG	876,00	308,91
RCN2	10344,46	3983,97
SEC23B	1420,95	742,30
SEL1L	9,87	3,91
SSR1	76,99	35,24
UGGT1	47,92	33,95
UGGT2	1633,64	1040,39
XBP1	260,37	129,23

Table S1. MiaPaCa2 cells siCtrl and siNUPR1 gene expression of proteins related with ER-stress defense pathways.

Gene name	log2FoldChange	p-value
Agr2	-1.29492	1.493e-05
Hyou1	-0,81965	0,00039
Hspa5	-0,75859	0,00052
Dnajc1	-0,72605	0,00065
Pdia4	-0,87943	0,00080
Dnajb9	-0,81762	0,00156
Sec11c	-0,40698	0,00163
Herpud2	-0,75244	0,00177
Pdia3	-0,58187	0,00304
Atf6	-0,59943	0,00542
Erp44	-0,41598	0,00653
Ssr1	-0,51263	0,00729
Man2a1	-0,52528	0,00828
Sel1l	-0,41316	0,01012
Ssr2	-0,42123	0,01176
Hspa13	-0,62219	0,01211
Dnajc22	-0,66955	0,01370
Lman1	-0,46967	0,01439
Uso1	-0,51128	0,01503
Edem3	-0,56645	0,01694
Xbp1	-0,26568	0,02001
Mlec	-0,26721	0,02685
Sec23b	-0,50919	0,03098
Ssr3	-0,40138	0,03900
Pdia6	-0,37603	0,04341
Dnajb14	-0,47681	0,04420
Canx	-0,30906	0,04494
Dnaja1	-0,41550	0,04755
Uggt1	-0,34831	0,04972

Table S2. Nupr1$^{+/+}$ and Nupr1$^{-/-}$ mice gene expression of proteins related with ER-stress defense pathways.

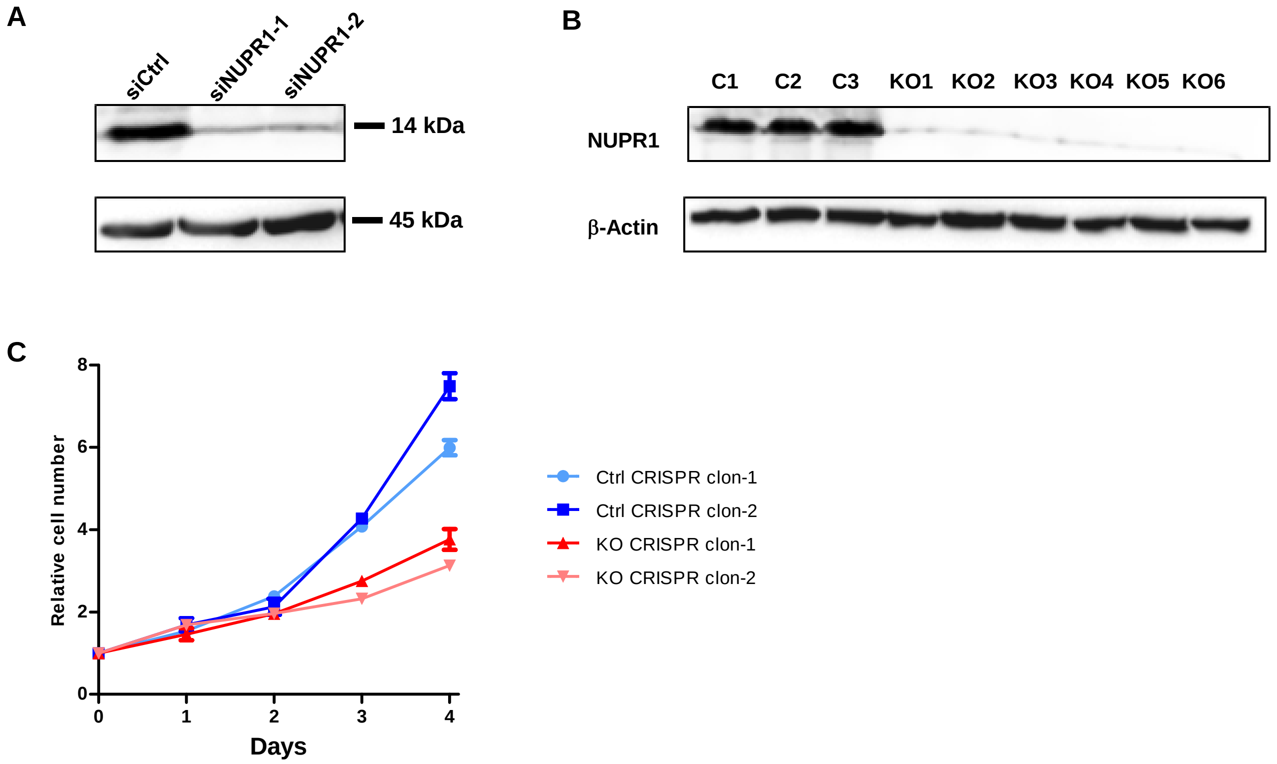

Figure S1

NUPR1 protein expression. Western blot analysis was performed in MiaPaCa2 cells transfected with siCtrl and siNUPR1-1 and siNUPR1-2 (A) and in 3 control and 6 CRISPR-Cas9 *NUPR1* knockout Panc-1 clones (B) to evaluate the NUPR1 protein levels. Relative grown of 2 control and 2 CRISPR-Cas9 NUPR1 knockout Panc-1 clones are shown (C).

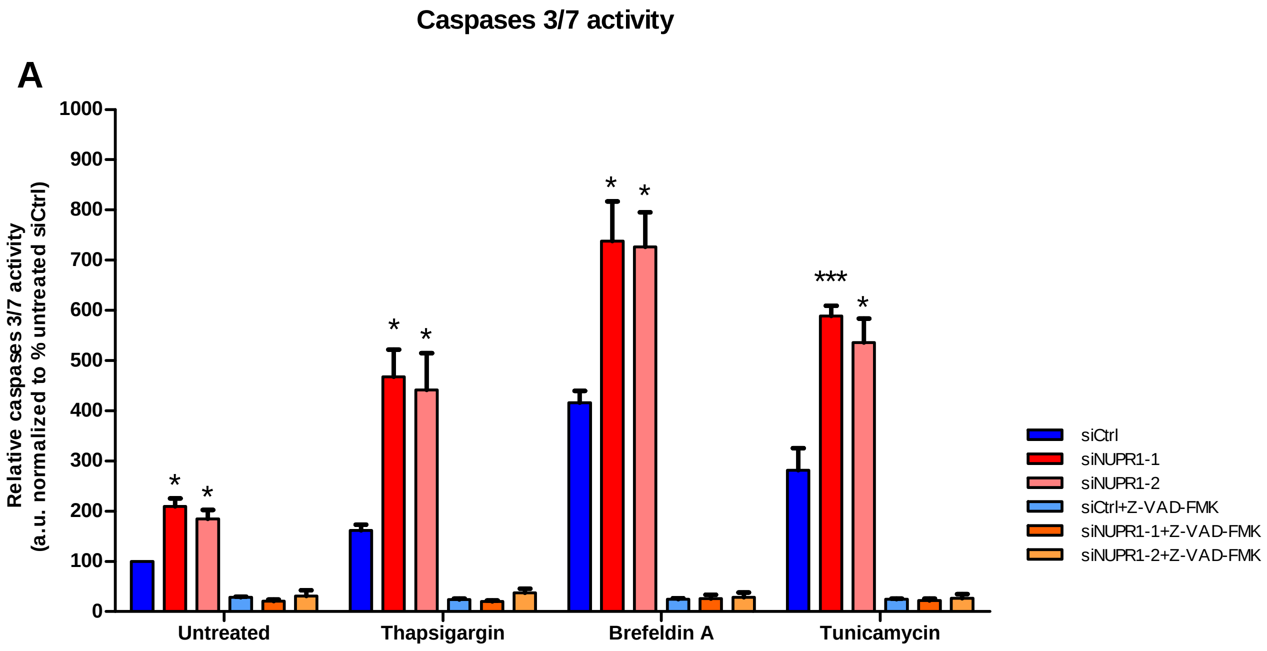

Figure S2

Nupr1 protects from apoptosis in stress situations *in vitro*. MiaPaCa2 cells were transfected with siCtrl or two different siNupr1 for 48 h. Cells were then incubated with thapsigargin, brefeldin A, or tunicamycin at 1 µM, for another 24 h in the presence or not of Z-VAD-FMK (10 µM). Caspase 3/7 activity was measured; data values were normalized to the untreated siCtrl. For each treatment, statistically significant differences (*$p < 0.05$, **$p < 0.01$, ***$p < 0.001$.) from siCtrl are shown.

A

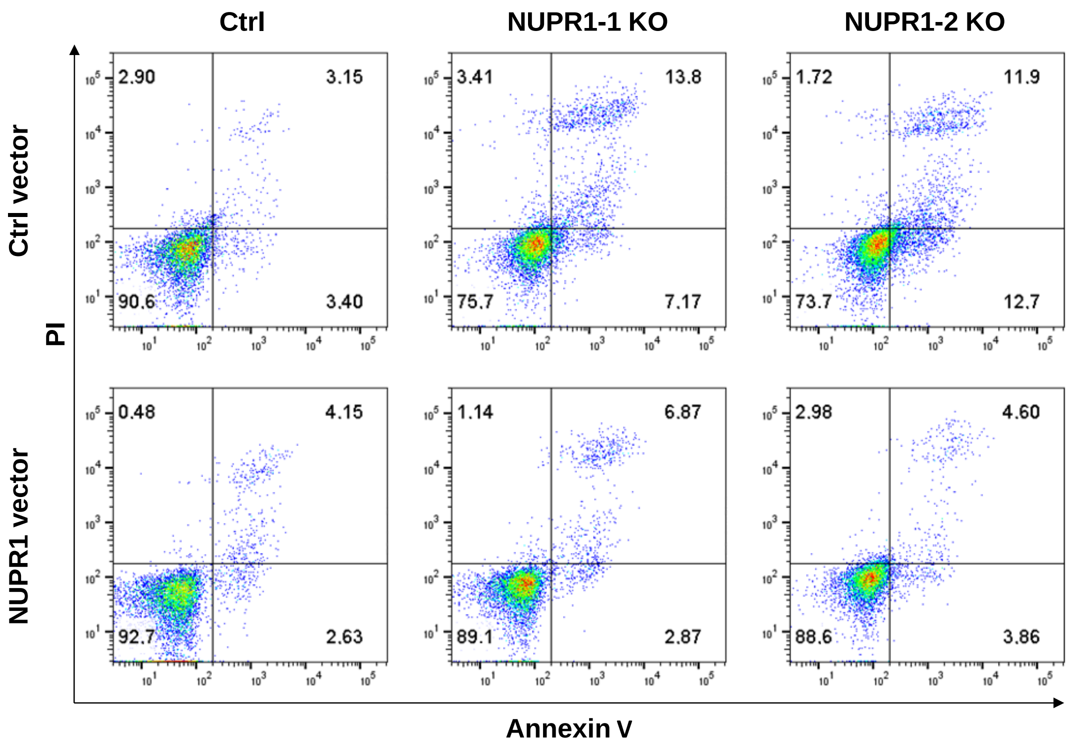

B

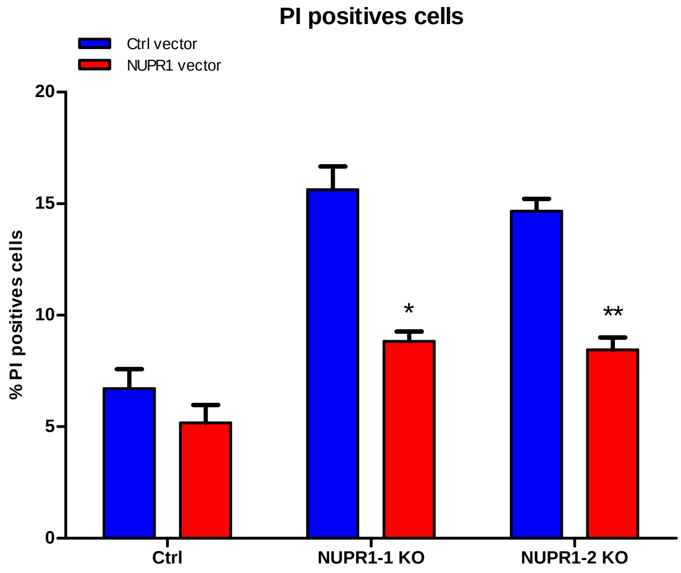

Figure S3

Reconstitution of NUPR1 expression in CRISPR-Cas9 knockout Panc-1 cells reduces cell death. Flow cytometry analysis was carried out after Annexin-V and PI staining in 2 independent Panc-1 *Nupr1* knockout clones, transfected with NUPR1-FLAG or a control vector. A representative experiment of the dot plot profile of cells was showed (A). The percentage of PI-positive cells (B) is showed. Data are means of triplicates ± SEM of 1 control and 2 *Nupr1* knockout clones. Statistically significant differences (*p < 0.05, **p < 0.01, ***p < 0.001.) are shown.

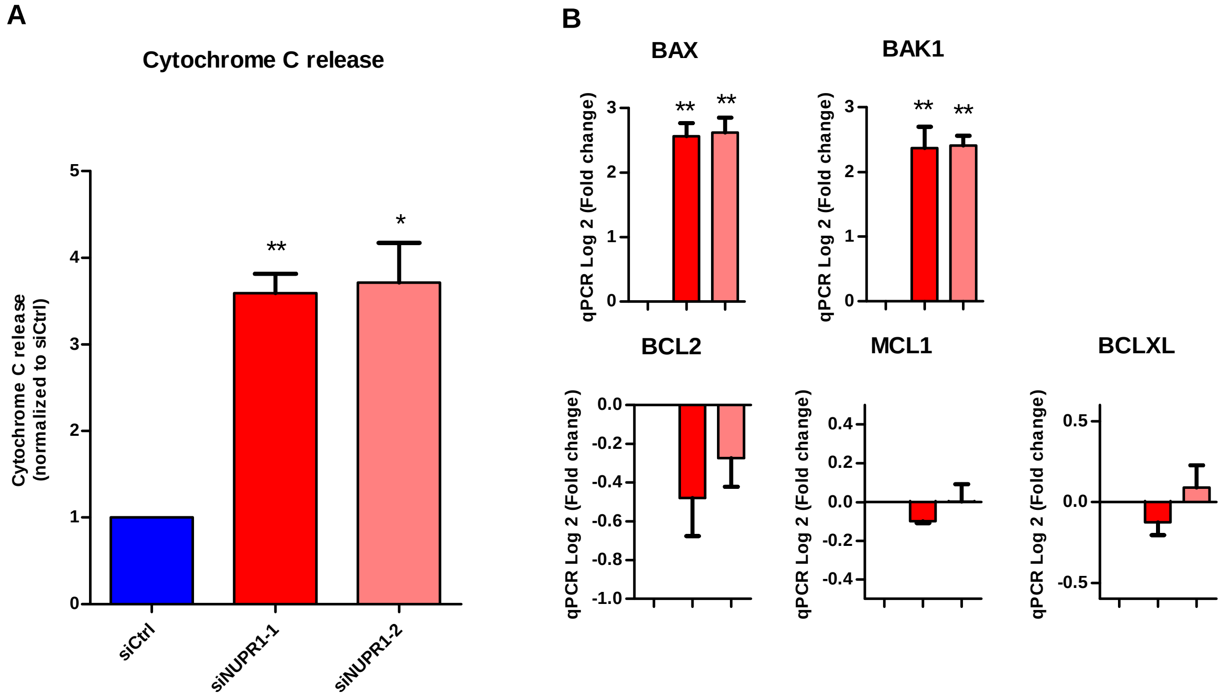

Figure S4

Nupr1 protects from cytochrome c release and BAK/BAX expression. Flow cytometry analysis of MiaPaCa2 cells transfected with siCtrl, siNUPR1-1, or siNUPR1-2 for 72 h was carried out using Cytochrome c Release Apoptosis Assay Kit for analysis of the cytochrome c release (A). Total RNA was extracted from MiaPaCa2 cells transfected with siCtrl, siNUPR1-1 or siNUPR1-2 for 72 h to evaluate the mRNA levels of Bcl-2 family genes of using RT-qPCR (B). Statistically significant differences from siCtrl (*p < 0.05, **p < 0.01, ***p < 0.001) was shown.

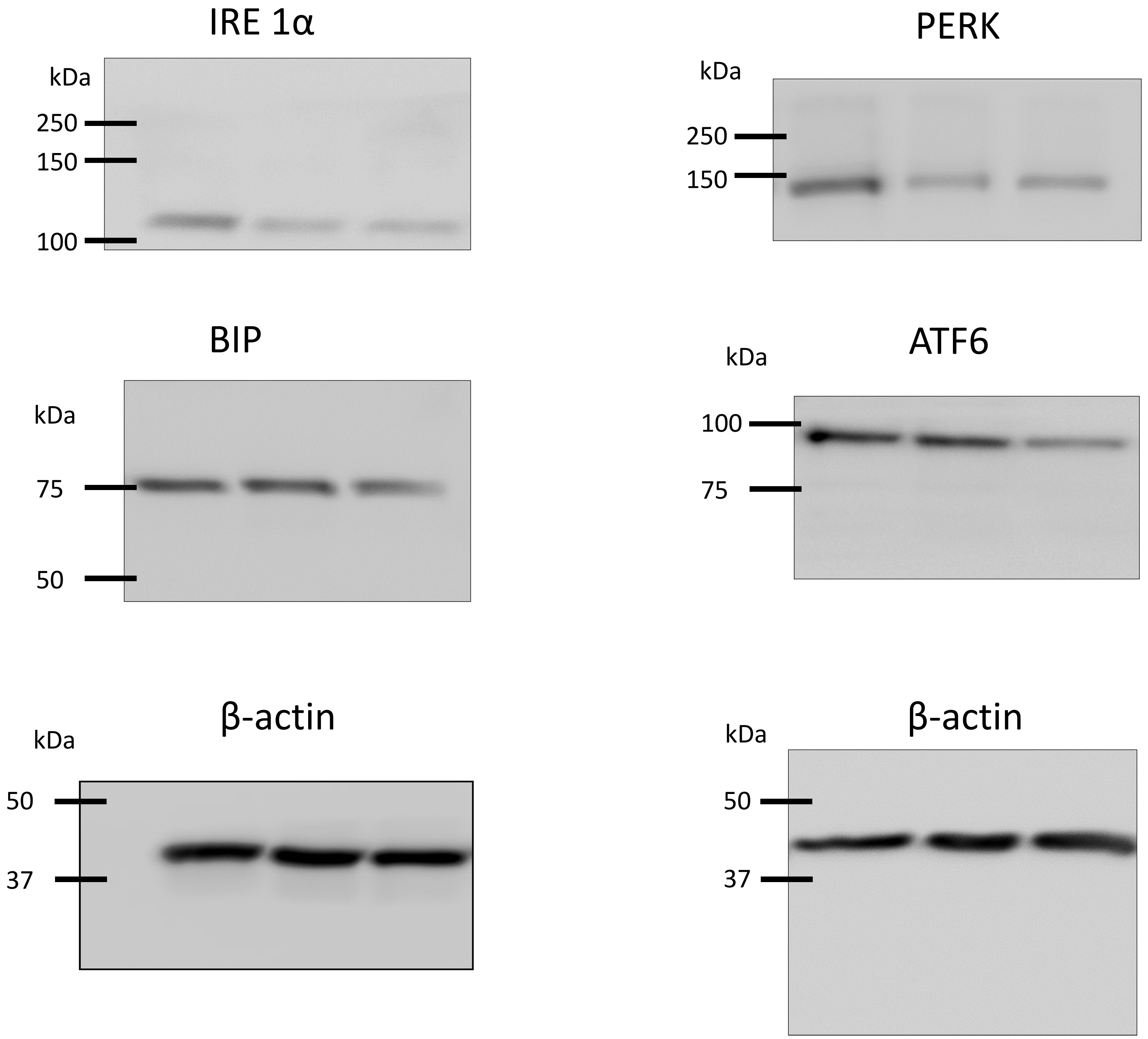

Full-length Western blot images

FIGURE S1

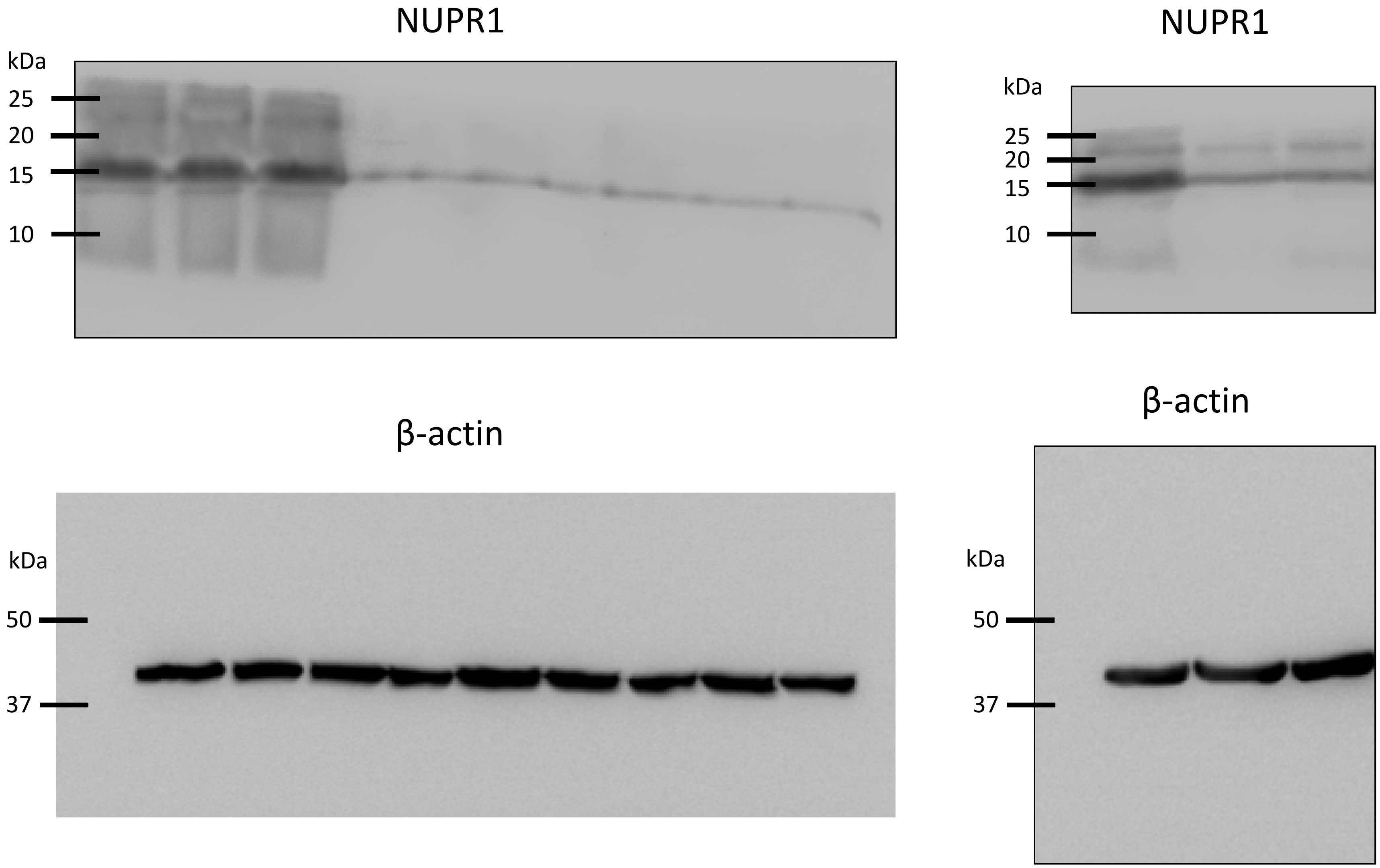

Full-length Western blot images
